# Patient-Completed Caprini Risk Score for Venous Thromboembolism Risk Assessment: Developed and Validated from 1,017 Medical and Surgical Patients

**DOI:** 10.1055/s-0042-1749170

**Published:** 2022-07-21

**Authors:** Zhu Zhang, Yifan Wu, Qingxia Liu, Fen Dong, Wenyi Pang, Kaiyuan Zhe, Jun Wan, Wanmu Xie, Wei Wang, Peiran Yang, Aihua Sun, Zhenguo Zhai

**Affiliations:** 1 Department of Pulmonary and Critical Care Medicine, Center of Respiratory Medicine, China-Japan Friendship Hospital, Beijing, People's Republic of China; 2 National Center for Respiratory Medicine, Beijing, People's Republic of China; 3 Institute of Respiratory Medicine, Chinese Academy of Medical Sciences, Beijing, People's Republic of China; 4 National Clinical Research Center for Respiratory Diseases, Beijing, People's Republic of China; 5 Department of Nursing, China-Japan Friendship Hospital, Beijing, People's Republic of China; 6 Institute of Clinical Medical Sciences, China-Japan Friendship Hospital, Beijing, People's Republic of China; 7 Institute of Basic Medical Sciences, Chinese Academy of Medical Sciences, Peking Union Medical College, Beijing, People's Republic of China

**Keywords:** venous thromboembolism, risk assessment, Caprini, self-report, validation

## Abstract

**Background** The Caprini Risk Score (CRS) is one of the most widely used risk assessment models for venous thromboembolism (VTE). A well-validated patient-completed CRS form may allow patients to self-report and simplify the evaluation by health care workers.

**Methods** The Chinese version of the CRS was optimized for easy understanding in a pilot study. The amended CRS form was completed by prospectively recruited patients and blinded nurses. The agreement levels of the individual questions and the total scores of patient and nurse-completed forms were compared using the Kappa value. The total scores were used for risk stratification of patients. Correlation and differences between patient and nurse-completed forms were analyzed using the Spearman correlation and Bland–Altman method, respectively.

**Results** We recruited 504 medical patients and 513 surgical patients, aged 52.7 ± 16.3 years, of which 443 (43.6%) were men, and 91.6% of the patients were educated beyond junior high school. The patients spent less time to complete the form compared with trained nurses. There was good question-to-question agreement between patient and nurse-completed CRS ( *k* >0.6 for most questions, *p* < 0.0001). The total scores also showed good agreement ( *k* = 0.6097, *p* < 0.0001), and enabled the classification of patients into different risk groups. The patient and nurse-derived scores were highly correlated (Spearman's *r* = 0.84), and without extreme values ( *p* < 0.0001).

**Conclusion** We have created and verified a Chinese version of the patient-completed CRS, which showed good agreement and correlation with nurse-completed CRS. CRS represents a suitable tool for VTE risk assessment of hospitalized patients in China.

## Introduction

Venous thromboembolism (VTE), including pulmonary thromboembolism (PTE) and deep venous thrombosis (DVT), is a common postoperative complication and a major cause of unexpected death in hospitalized patients. 1 Nearly half of the VTE cases are associated with hospitalization, which is a major triggering factor for VTE. 2 3 VTE is the cause of death in up to 2.1% of hospitalized patients in China. 4 Approximately 20 to 50% of patients who survive VTE develop post-thrombotic syndrome, which may lead to venous ulcers, and 2 to 4% of patients with PTE develop chronic thromboembolic pulmonary hypertension (CTEPH), which is potentially fatal. 5 These conditions have a negative effect on the quality of life of individuals and heavily burden the health care system. 6 Therefore, the appropriate prophylaxis of VTE is highly important for the safety of hospitalized patients and for avoiding the sequelae of VTE. 7

The Caprini Risk Score (CRS) is the most widely used risk assessment model (RAM) for VTE in clinical practice and has been validated in medical patients and surgical patients worldwide. 8 9 The ninth edition of the American College of Chest Physicians (ACCP) Antithrombotic Therapy and Prevention of Thrombosis guidelines has recognized that the Caprini RAM can be used for stratification of non-orthopaedic surgical patients. 10 However, the current CRS assessment is mainly completed by health care workers such as doctors and nurses, which is accurate, but may be time consuming. 11 To increase the level of knowledge, involvement, and compliance of patients, 12 13 a patient completed CRS in English was created in 2017. 14 Thus far, the patient-completed CRS has been translated into Spanish, Polish, and Arabic, and its effectiveness has been verified in those versions. 15

The Chinese version of the patient-completed CRS was constructed earlier but was verified only in a small group of patients. 16 It lacks external validation and application in clinical practice. Therefore, the present study aimed to test the reliability and validity of the Chinese version of patient-completed CRS in a large number of hospitalized patients and to evaluate whether this RAM can be widely used in clinical practice to replace the assessment by health care workers.

## Methods

### Study Design

We conducted a prospective, observational, single-center study and recruited 514 inpatients from the surgical units and 503 inpatients from the medical unit between January 1 and December 31, 2019. The inclusion criteria were as follows: (1) patients older than 18 years; (2) patients with first admission to the medical and surgical units within 24 hours; and (3) willingness to participate in the study. The exclusion criteria were as follows: (1) patients with a diagnosis of PTE or DVT on admission; (2) patients with cognitive impairment; (3) inability to read or write; (4) patients with mental diseases; (5) patients with visual impairment; and (6) refusal to cooperate. Our work has been reported in line with the STARD (Standards for the Reporting of Diagnostic accuracy studies) criteria.

### Construction and Validation of the Patient-Completed CRS

The English patient-completed CRS was translated into Chinese by two independent experts with good English proficiency and professional medical knowledge. Then, the translated version was reviewed by three experts with rich clinical experience in VTE to ensure its accuracy.

To identify the ambiguous and misinterpreted areas of the Chinese version, a pilot experiment was conducted, in which 100 patients admitted to the Department of Pulmonary and Critical Care Medicine in our hospital were consecutively recruited using the inclusion and exclusion criteria. The patients were asked to complete the form and provide feedback, which were used to adjust the expression of the context and the order of questions to help Chinese patients understand the form better ( Supplementary Fig. S1 ).

Patients were prospectively recruited from internal medicine and surgery departments by a continuous inclusion and convenience sampling method. The patients and trained nurses were asked to complete the revised Chinese version of the CRS form 24 hours after admission to hospital. The patients did not undergo any intervention during their evaluation process (NCT04663477).

### Statistical Analysis

Agreement between patient-completed CRS and trained nurse-completed CRS was measured using Cohen's Kappa value. The scores were summed up and Spearman's coefficient was used to assess the overall correlation between patient and nurse-derived total scores. The Bland–Altman method was used to evaluate the differences between the scores calculated by the patients and those by the nurses. The overall CRS was categorized into very low (0), low (1–2), moderate (3–4), and high risk (≥5) based on the ninth ACCP guidelines, and the agreement level between patient-completed and nurse-completed CRS was measured using Kappa values. All statistical analyses were conducted using SAS 9.4 (SAS Institute Inc., Cary, North Carolina, United States). Data were expressed as the composition ratio and mean ± standard deviation (SD). All *p* -values were calculated using two-sided tests, and significance was determined a priori at the 0.05 level.

## Results

### Characteristics of the Subjects

In total, we recruited 1,017 patients to complete the CRS. Among them, 504 were medical patients and 513 were surgical patients. Mean (± SD) patient age was 52.7 ± 16.3 years, and 443 (43.6%) were men. Most of the participants were well educated and lived in the city ( Table 1 ). Medical patients were from pulmonary critical care medicine, gastroenterology, cardiology, rheumatology, neurology and surgical patients were from gynecology, orthopaedics, general surgery, etc. ( Table 2 ). The median time for patients to complete the form was 4 minutes (3–6 minutes), which was less than the time needed by trained nurses.

**Table 1:** Demographic information of the patients ( *n* = 1,017)

Characteristic	Surgical patients ( *n* = 513)	Medical patients ( *n* = 504)	Total ( *n* = 1017)
Age			
Mean ± SD	49.4 ± 16.0	56.0 ± 16.0	52.7 ± 16.3
Sex			
Male	187 (18.3)	256 (25.2)	443 (43.6)
Female	326 (32.1)	248 (24.4)	574 (56.4)
Educational level			
Master degree	29 (2.9)	26 (2.6)	55 (5.4)
Bachelor degree	221 (21.7)	177 (17.4)	398 (39.1)
Junior and senior high school	228 (22.4)	251 (24.7)	479 (47.1)
Primary school and below	35 (3.4)	50 (4.9)	85 (8.4)
Comorbid disease			
Hypertension	116 (11.4)	145 (14.3)	261 (25.7)
Diabetes	48 (4.7)	72 (7.1)	120 (11.8)
Coronary heart disease	29 (2.9)	66 (6.5)	95 (9.3)
Cervical spondylosis	41 (4.0)	43 (4.2)	84 (8.3)
Arthritis	19 (1.9)	21 (2.1)	40 (4.0)
Others	73 (7.2)	101 (10.0)	174 (17.1)
None	285 (28.0)	204 (20.1)	489 (48.1)

Abbreviation: SD, standard deviation.

**Table 2:** Distribution of included patients' departments

Surgery or Medical Department type	No. (%)
Medical Departments	
Pulmonary critical care medicine	307 (30.2)
Gastroenterology	84 (8.3)
Cardiology	73 (7.2)
Rheumatology	20 (2.0)
Neurology	20 (2.0)
Surgical Departments	
Gynecology	142 (14.0)
Orthopaedics	98 (9.6)
General surgery	83 (8.2)
Ear-nose-throat	63 (6.2)
Urology	50 (4.9)
Thoracic surgery	49 (4.8)
Cardiovascular surgery	28 (2.8)

### Question-to-Question Agreement between Patient-Completed and Nurse-Completed CRS

Cohen's Kappa values were used to measure the extent of agreement between patients' and health care providers' assessments. The vast majority of the items of the scale showed good agreement between the two groups, except for “one surgery-related item,” and the Cohen's Kappa values ranged from 0.63 to 1.0. The corresponding data are presented in Table 3 . High inter-rater reliability was found for all items, and the Kappa values for all items were statistically significant (all *p* -values <0.0001). Furthermore, the items on the evaluation scale showed consistency between the patient-evaluated and nurse-evaluated scores. The Kappa values are all statistically significant after testing ( *p* -value <0.0001) ( Table 3 ). The degree of agreement was evaluated based on the Kappa value (Kappa ≤0.4 indicated low consistency, 0.4< Kappa≤ 0.6 indicated moderate consistency, 0.6< Kappa ≤0.8 indicated high consistency, 0.8 <Kappa ≤1 indicated high consistency). The vast majority of items showed Kappa values> 0.6, except for elective surgery.

**Table 3:** Question-to-question agreement level between patient-completed and nurse-completed forms ( *n* = 1,017)

Item	Kappa	*p*
1. Age		
1.1	1.0000	<0.0001
1.2	0.9772	<0.0001
1.3	0.9878	<0.0001
1.4	0.9804	<0.0001
2. Add 1 POINT for each statement that applies to you		
2.1	0.7082	<0.0001
2.2	0.6432	<0.0001
2.3	0.7733	<0.0001
2.4	0.7632	<0.0001
2.5	0.8630	<0.0001
2.6	0.6301	<0.0001
2.7	0.8153	<0.0001
2.8	0.9076	<0.0001
3. For WOMEN ONLY, add 1 POINT for each statement that applies to you		
3.1	0.8786	<0.0001
3.2	0.9226	<0.0001
3.3	0.7991	<0.0001
4. Add 2 POINTS for each statement that applies to you		
4.1	0.7733	<0.0001
4.2	0.6362	<0.0001
4.3	0.6767	<0.0001
5. Add 3 POINTS for each statement that applies to you		
5.1	0.7733	<0.0001
5.2	0.6362	<0.0001
5.3	0.6767	<0.0001
6. Please select points for each statement that applies to you		
6.1	0.7717	<0.0001
6.2	0.8508	<0.0001
7. Add 5 POINTS for each statement that applies to you		
7.1	0.8567	<0.0001
7.2	0.8325	<0.0001
7.3	0.8567	<0.0001
7.4	1.0000	<0.0001
8. If you have a SCHEDULED SURGERY coming up, please select an option		
8.1	0.4285	<0.0001
8.2	0.6036	<0.0001

### Agreement Level of Risk Stratification between Patient-Completed and Nurse-Completed CRS

**Fig. 1:**
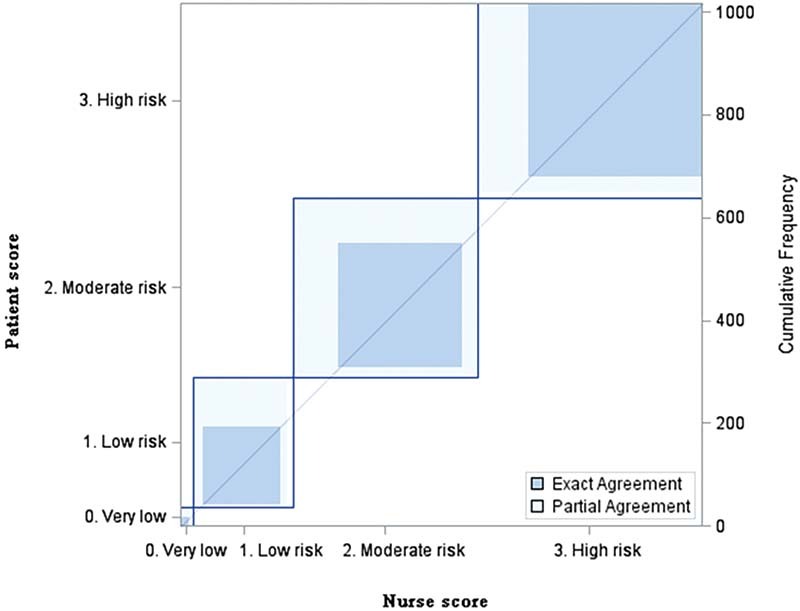
Agreement level in risk stratification between nurse-completed and patient-completed forms ( *n* = 1017). The figure indicated that the risk stratification of patient self-evaluation and nurse-completed evaluation was consistent.

We added up the patient-completed and nurse-completed CRS and categorized them into very low, low, medium, and high risk according to the ninth ACCP guidelines. We found that there were 18 (1.8%) patients with very low risk, 152 (15.0%) patients with low risk, 242 (23.8%) patients with medium, and 339 (33.3%) patients with high risk in both patient-completed and nurse-completed risk stratification. The Kappa value was 0.6097 ( *p* < 0.0001), as shown in Table 4 and Fig. 1 . The Kappa value was 0.6097 ( *p* < 0.0001), indicating that the risk stratification of patient self-evaluation and nurse-completed evaluation was consistent.

**Table 4:** Agreement level by stratification between patient-completed and nurse-completed Caprini risk score ( *n* = 1017)

Patient-completed No. (%)	Nurse-completed				Kappa	*p*
	Very low	Low	Moderate	High		
Very low	18 (1.8)	18 (1.8)	0 (0)	0 (0)	0.6097	<0.0001
Low	5 (0.5)	152 (15.0)	87 (8.6)	10 (1.0)		
Moderate	3 (0.3)	14 (1.4)	242 (23.8)	89 (8.6)		
High	0 (0)	10 (1.0)	30 (3.0)	339 (33.3)		

### Correlation and Difference between the Total Scores Calculated by Patient-Completed and Nurse-Completed CRS

**Fig. 2:**
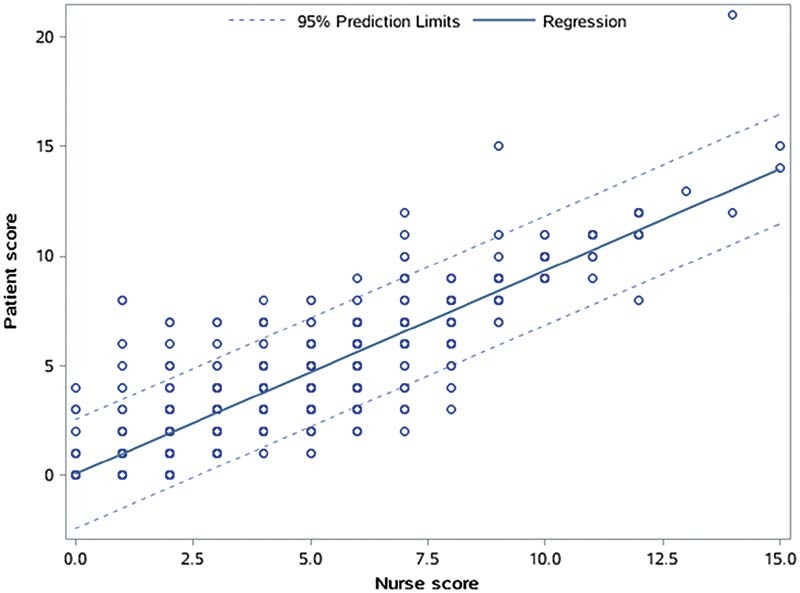
Correlation between patient-completed and nurse-completed Caprini risk score ( *n* = 1017). The Spearman correlation coefficient was 0.8415. There is no difference between the scores of patients' self-assessment and nurses' assessment.

**Fig. 3:**
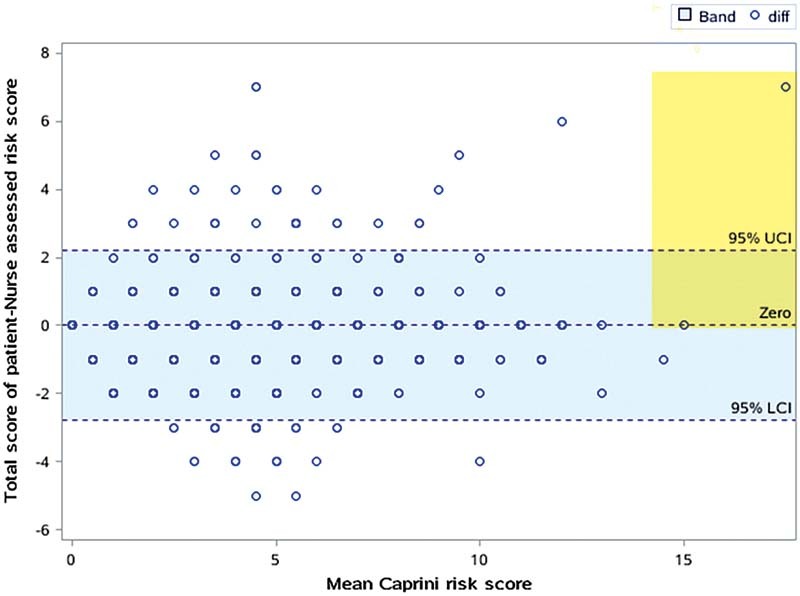
Bland-Altman plot of patient-completed and nurse-completed Caprini risk score ( *n* = 1017). No trend for extreme values were found through this figure.

The Spearman correlation and the Bland–Altman method were used to measure the correlation and difference between patient-completed and nurse-completed CRS. The Spearman correlation coefficient was 0.8415 ( *p* < 0.0001) ( Fig. 2 ), and we did not find any trend for extreme values according to the Bland-Altman ( Fig. 3 ). There is no difference between the scores of patients' self-assessment and nurses' assessment.

## Discussion

We created and validated the Chinese version of CRS, which is the most useful VTE RAM, and showed good correlation and agreement between patient-filled and nurse-filled forms. Our study may facilitate the use of the VTE RAM in China and promote the engagement of patients in the prevention of thrombosis.

Most clinical evaluations are performed by medical staffs, which are accurate but time-consuming. For this reason it has been suggested to limit the use of these scoring models in the clinical setting. 11 Additionally, this limits the involvement of patients in the assessment process. In contrast, patient-reported questionnaires have been found effective and well suited when complex health issues are explored in medical and surgical patients. 17 18

CRS is the most widely used risk assessment tool and has been approved for risk-stratification, including very low-risk, low-risk, moderate-risk and high-risk categories, according to the ninth ACCP guidelines. 9 High-risk patients are individuals with scores above 5, indicating a sub-group of patients at great risk for VTE. The high-risk patients may need preventive anticoagulant treatment. According to the guidelines, 9 the set point of high risk is 5, but this has since been found to vary by study population. Specifically, the set point of highest risk is 9 for patients having general surgery, 19 10 for patients with total joint arthroplasty, 20 11 for patients undergoing colorectal cancer surgery, 21 and 12 for patients following hip fracture. 22 In our study, nearly 27% of participants scoring above 5 were categorized as high risk. More importantly, as the VTE risk escalates with each point increase, we reasoned that there may not be a fixed and universal set point in VTE risk assessment. Therefore, we believe that it would be informative to report the number of patients different scores. The numbers (%) of patients with score above 5, 6, 7, 8, 9, and 10 were 278 (27.34%), 168 (16.52%), 67 (6.59%), 46 (4.52%), and 26 (2.56%), respectively.

In 2017, Fuentes et al 14 were the first to publish the English version of patient-completed CRS and reported good agreement between patient and physician-completed CRS. With this method, only three metrics remain to be assessed by health care providers. One year later, Paz Ros 15 translated the English version of patient-completed CRS into Spanish, Arabic, and Polish and found similar results in BMI. Chen et al 16 tested a Chinese version of the patient-completed CRS in a small group of patients and found good correlation and agreement levels. However, in 2019, Veith et al 23 compared patient-completed and physician-completed CRS in plastic surgery patients and found that reliance on patient-completed scores alone would promote nearly 25% of patients receiving inappropriate prophylactic strategies. In their study, almost 50% of the risk factors showed disagreement between patient- and health provider-completed forms ( Table 5 ). Taken together, these studies show that whether the CRS can be completed by patients instead of health care workers is controversial, and further external validation using a larger number of patients is needed in for the Chinese version. Therefore, we created and validated the Chinese patient-completed CRS according to the guidelines of the International Society for Pharmacoeconomics and Outcome Research (ISPOR). According to the results of our pilot study and original authors' suggestions for revision, we modified sections 4.1, 5.1–5.3, 7.1, and 7.3 of the questionnaires to help Chinese patients understand it easily.

**Table 5:** Summary of the different versions of patient-completed Caprini risk score and their evaluation

No.	Title	Study design	Statistical methods	Major findings: agreement between patient-completed and physician-completed CRS for cumulative score	Reference
1	Validation of a Patient-Completed Caprini Risk Score for Venous Thrombo-embolism Risk Assessment.	The study was divided into three phases: (1) Created a patient-completed Caprini risk score (CRS) form; (2) Conducted a pilot study on 20 medical or surgical patients to refine the CRS document; (3) Validated the CRS form within 42 medical or surgical patients. The work was conducted at the John Stroger Jr. Hospital from October 2016 through January 2017.	Cohen's Kappa value, linear correlation (Spearman's correlation coefficient), and the Bland–Altman test was used.	(1) Cohen's Kappa value: (a) ACCP criteria: 0.9 (95% CI: 0.73–1.00); (b) CRS above 8: 0.8 (95% CI: 0.65–0.85); (c) CRS above 11: 1.00 (SD: 0.00). (2) Spearman's correlation coefficient: 0.95 ( *p* < 0.01). (3) Bland–Altman plot did not show trends for extreme values.	Fuentes, et al TH Open. 2017. 14
2	Validation of a Patient-Completed Caprini Risk Assessment Tool for Spanish, Arabic, and Polish Speakers	The study was divided into three phases: (1) Translated the patient-completed CRS from English to Spanish, Arabic, and Polish; (2) Conducted a pilot study on 83 medical or surgical patients to identify additional challenges specific to each language; (3) Validated the CRS form among 129 medical or surgical patients. The study was conducted at the John H. Stroger Hospital from October 2016 throughMarch 2017.	The Cohen's Kappa value, linear correlation (Spearman's correlation coefficient), and the Bland–Altman test were used.	(1) Cohen's Kappa value: 0.93 (1.00, 0.93 and 0.85 for Spanish, Arabic and Polish versions, respectively). (2) Spearman's correlation coefficient: 0.97 (0.98, 0.95 and 0.99 for Spanish, Arabic and Polish versions, respectively; all *p* < 0.01). (3) Bland–Altman test did not show trend for extreme values.	Paz Rios et al Clin Appl Thromb Hemost. 2018. 15
3	Clinical Validation of the Chinese Version of Patient Completed Caprini Risk Assessment Form	The study was divided into three main steps: (1) Translated the patient-completed CRS into Chinese; (2) Conducted a pilot study on 10 medical or surgical patients; (3) Validated the CRS form in 70 internal medical inpatients and 70 surgical inpatients. The study was conducted in Beijing Shijitan hospital from January 2019 to January 2020.	The Cohen's Kappa value, linear correlation (Spearman's correlation coefficient), and the Bland–Altman test were used.	(1) Cohen's Kappa value: (a) Patient-completed vs. physician-completed CRS: 0.76 ( *p* < 0.0001); (b) final value of physician-completed CRS (physician-completed CRS plus body mass index) vs. CRS in EMR: 0.97 ( *p* < 0.0001). (2) Spearman's correlation coefficient: (a) Patient-completed vs. physician-completed CRS: 0.978 ( *p* < 0.0001); (b) final value of physician-completed CRS (physician-completed CRS plus body mass index) vs. CRS in EMR: 0.994 ( *p* < 0.0001). (3) Disagreement rate derived from Bland–Altman test: (a) Patient-completed vs. physician-completed CRS: 3.57%; (b) final value of physician-completed CRS (physician-completed CRS plus body mass index) vs. CRS in EMR: 1.43%.	Chen et al Clin Appl Thromb Hemost. 2020. 16
4	Direct Comparison of Patient-completed and Physician-completed Caprini Scores for Plastic Surgery Patients	50 plastic surgery patients were recruited for a previous patient-completed CRS validation from 2 plastic surgery clinics at the University of Utah Hospital and Huntsman Cancer Institute from August 2018 to October 2018.	The Cohen's Kappa value, linear correlation (Spearman's correlation coefficient), the Bland–Altman test and Wilcoxon rank-sum test were used.	Spearman's correlation coefficient: 0.694 ( *p* < 0.0001).	Veith et al Plast Reconstr Surg Glob Open. 2019. 19

Abbreviation: ACCP, American College of Chest Physicians; CI, confidence interval; EMR, electronic medical record; SD, standard deviation.

We measured the question-to-question agreement level between patient-completed and nurse-completed CRS. Our results showed that there was good agreement in all questions except one: “I have a scheduled surgery under general or regional anesthesia for less than 45 minutes.” The Kappa value of that question was 0.4285, while Kappa values of all other questions ranged from 0.6036 to 1.000 (all *p* -values <0.0001), which is similar to the range reported by Fuentes et al 14 We think that this may be related to the lack of professional medical knowledge of patients so that they were likely to inaccurately estimate the length of the operation time. Hence, according to our study, we indicated that trained nurses should assess the question with patients to determine the accuracy of that question. It is worth noting that our study showed good agreement for the question “age” and the sub-questions of “For women only, add 1 point for each statement that applies to you,” the Kappa values of these questions were nearly 1.000 and 0.8786, 0.9226 and 0.7997, indicating that medical staff did not need to assess these questions again. It is essential to evaluate the risk of thrombosis of patients and select optimal prophylactic strategy. 24 Our study showed good agreement in risk stratification according to the total scores calculated by patient and nurse-filled form, which can help clinicians select correct anticoagulation methods.

The strength of our study was that our study did an external validation of Chinese patient-completed CRS, and so far our study was the largest and the most convincing. We conducted a pilot experiment and optimized the CRS using the results obtained from 100 patients. Then we continuously enrolled a large number of patients from internal medicine and surgery departments. The limitation of this research was that this was a prospective, observational, single-center study; in the future, we plan to conduct a multicenter study. Additionally, our patients are generally highly educated, as nearly half of them had a college degree or higher, which may affect the usefulness of the form in less well-educated patients.

In conclusion, we adapted and revised the English version of the patient-completed CRS into Chinese and validated its effectiveness in a large group of patients. Our study showed that there is a high agreement level and good correlation between patient-completed and trained nurse-completed CRS. We conclude that the Chinese patient-completed CRS is a suitable tool for VTE risk assessment in hospitalized patients in China.

## Highlights

1. Caprini Risk Score (CRS) is usually completed by health workers in a time-consuming process.
2. English version of patient-completed CRS has been validated to have good agreement with physician-completed CRS.
3. Patient-completed CRS has been developed in Chinese and validated in 1,017 patients to simplify CRS completed by trained nurses.

## Notes

**What is Known on This Topic?**

1. Caprini Risk Score (CRS) is usually completed by health workers in a time-consuming process.
2. English version of patient-completed CRS has been validated to have good agreement with physician-completed CRS;

**What Does This Paper Add?**

1. Patient-completed CRS has been developed in Chinese and validated in 1,017 patients to simplify CRS completed by trained nurses.
2. Patient-completed CRS represents a suitable tool for VTE risk assessment of hospitalized patients in China.
