# Supplementary material for: Patient-Completed Caprini Risk Score for Venous Thromboembolism Risk Assessment: Developed and Validated from 1,017 Medical and Surgical Patients
Source: TH Open. 2022 Jul 21;6(3):e184–93. doi: 10.1055/s-0042-1749170 (PMC9303073; doi:10.1055/s-0042-1749170)
Supplement: Supplementary file 1 — Supplementary Material [file 10-1055-s-0042-1749170-s210078.pdf]

您存在深静脉血栓形成（DVT）的风险吗？（患者版）

深静脉血栓形成(DVT)是指在您下肢的深静脉内有血栓形成，只有您的医生可以评估您深静脉血栓形成的风险。通过回顾您的个人病史和目前的健康状况，有助于判断您是否有罹患这种疾病的风险。为了您与您爱的人，请花费一定时间完成这个表格。之后务必与您的医生交流，了解您的深静脉血栓形成风险情况与预防措施。

1. 请选择您的**年龄**：

0-40 岁(0 分)☐ 41-60 岁(1 分) ☐ 61-74 岁(2 分) ☐ ≧75 岁（3 分） 分数：

2.每一条适用于您的情况加 **1 分**:

☐ 在过去的一个月里，我**接受了全身/局部麻醉手术**，手术时间持续 45 分钟以上。
☐ 在过去的一个月里，我有过**下肢静脉曲张**（或目前仍有）症状。
☐ 在过去的一个月里，我有过**下肢肿胀**（或目前仍有）症状。
☐ 在过去的一个月里，我有过**心脏发病作**。
☐ 在过去的一个月里，我有过**严重的感染**，并住院治疗，例如肺炎、蜂窝织炎等，或目前仍有上述情况。
☐ 我曾经有过**炎症性肠病史**(主要包括克罗恩病或溃疡性结肠炎)。
☐ 我曾经有过（或目前仍患者有）**心力衰竭**。
☐ 我有**慢性肺病病史**(例如慢性阻塞性肺疾病、肺气肿、慢性气管炎)，但不包括哮喘。 分数：

3.以下选项仅对**女性**有效，对您适用的每种情况加 **1 分**:

☐ 我近 1个月内正在使用避孕措施（口服避孕药，皮下埋植剂，激素贴片，含激素的宫内节育器，长效避孕药）或激素替代治疗。不包括安全套或屏障避孕方法。
☐ 我目前正在怀孕或在1个月内有生产史。
☐ 我曾经有过不明原因的死胎史,3 次以上的自然流产史、子痫前期合并早产,或小于胎龄儿(低出生体重儿)。
分数：

4.每条适用于您的情况加 **2 分**:

☐ 在过去的 6 个月里，我的医生告诉我患有癌症、白血病、淋巴瘤或黑色素瘤。
☐ 在过去的 1 个月里，我曾经有过下肢的石膏固定，使我不能弯曲和/或正常行走。
☐ 在过去的 1 个月里，医生或护士曾经在我的颈部或胸部放置了 PICC 穿刺针、输液港或中央静脉置管，目的是进行输血或用药（或者目前仍有上述情况）。 分数：

5.每条适用于您的情况加 **3 分**:

☐ 我的下肢、上肢、腹部或肺部有静脉血栓病史。**（脑梗塞、心肌梗死除外）**
☐ 我的亲属(父母、祖父母、阿姨、叔叔、兄弟姐妹、堂兄弟姐妹)是否有上/下肢、腹部或肺部静脉血栓病史。**（脑梗塞、心肌梗死除外）**
☐ 您或与您有血缘关系的亲属是否曾经被告知存在**血液检测异常（例如抗凝血酶缺陷症，蛋白C或S缺乏，LeidenV因子、凝血酶原G20210A突变，抗磷脂抗体综合征等）**，提示血栓发生的风险增加。

6.请选择以下适用于您的情况：

☐ 我由于单次步行距离不能超过 9 米而卧床，时间少于 3 天 (**1 分**)。
☐ 我由于单次步行距离不能超过 9 米而卧床，达 3 天或以上 (**2 分**)。 分数：

Supplementary Fig. S1 Patient-completed Caprini risk score (Chinese questionnaire).

 © 2022. The Author(s).

7.每条适合于您的情况加 **5 分**:

- ☐ 在过去的一个月里，我接受了髋关节或膝关节置换手术。
- ☐ 在过去的一个月里，我曾经有过臀部、骨盆和腿部骨折。
- ☐ 在过去的一个月里，我曾经经历过严重的创伤(例如由于跌倒或车祸造成的**两处及以上骨折**)。
- ☐ 在过去的一个月里，我曾经得过中风（脑血栓或脑出血，或短暂的脑缺血发作)。

分数：

8.如果您将接受择期手术，请选择一个选项。

- ☐ 我**将接受**一个全身或局部麻醉下的**择期手术**，预计手术时间少于 45 分钟 (**1 分**)。
- ☐ 我**将接受**一个全身或局部麻醉下的**择期手术**，预计手术时间超过 45 分钟 (**2 分**)。

分数：

医疗保健--专业人员填写：

9.根据患者的体重指数，每项陈述增加 **1 分**:

- ☐ 超重(BMI> 25)。 BMI=体重(Kg)/身高^2(m)

分数：

10.每增加一个风险因素加 1 分: (这些危险因素尚未经过验证研究，但已在文献中显示与血栓形成有关)

- ☐ 病态肥胖(BMI >40)
- ☐ 吸烟(1 个月内)
- ☐ 糖尿病患者需要注射胰岛素
- ☐ 化疗/放疗(6 个月内)
- ☐ 输血(3 个月内)
- ☐ 人类免疫缺陷病毒(HIV)

分数：

11. 与患者一起对血栓风险进行重新评估，包括以下内容，并根据需要调整评分:

- ☐ 评估患者下肢肿胀情况。包括任何类型的凹陷性水肿。
- ☐ 回顾女性患者的产科病史(参见第 3 条“仅针对女性”，表述 3)。
- ☐ 回顾患者血栓形成的家族史。包括一级亲属、二级亲属和三级亲属，包括浅静脉血栓形成和深静脉血栓形成。

调整分数:

总分数

Supplementary Fig. S1 (*Continued*)

 © 2022. The Author(s).
